# Effectiveness of Micronutrient Powders (MNP) in women and children

**DOI:** 10.1186/1471-2458-13-S3-S22

**Published:** 2013-09-17

**Authors:** Rehana A Salam, Ceilidh MacPhail, Jai K Das, Zulfiqar A Bhutta

**Affiliations:** 1 Division of Women & Child Health, The Aga Khan University, Karachi, Pakistan; 2 University of Toronto, Toronto, Ontario, Canada; 3 Global Child Health and Policy, Centre for Global Child Health, The Hospital for Sick Children, Toronto, ON, Canada

## Abstract

**Introduction:**

More than 3.5 million women and children under five die each year in poor countries due to underlying undernutrition. Many of these are associated with concomitant micronutrient deficiencies. In the last decade point of use or home fortification has emerged to tackle the widespread micronutrient deficiencies. We in this review have estimated the effect of Micronutrient Powders (MNPs) on the health outcomes of women and children.

**Methods:**

We systematically reviewed literature published up to November 2012 to identify studies describing the effectiveness of MNPs. We used a standardized abstraction and grading format to estimate the effect of MNPs by applying the standard Child Health Epidemiology Reference Group (CHERG) rules.

**Results:**

We included 17 studies in this review. MNPs significantly reduced the prevalence of anemia by 34% (RR: 0.66, 95% CI: 0.57-0.77), iron deficiency anemia by 57% (RR: 0.43, 95% CI: 0.35-0.52) and retinol deficiency by 21% (RR: 0.79, 95% CI: 0.64, 0.98). It also significantly improved the hemoglobin levels (SMD: 0.98, 95% CI: 0.55-1.40). While there were no statistically significant impacts observed for serum ferritin and zinc deficiency. Our analysis shows no impact of MNPs on various anthropometric outcomes including stunting (RR: 0.92, 95% CI: 0.81, 1.04), wasting (RR: 1.13, 95% CI: 0.91, 1.40), underweight (RR:0.96, 95% CI: 0.83, 1.10), HAZ (SMD: 0.04, 95% CI: -0.13, 0.22), WAZ (SMD: 0.05, 95% CI: -0.12, 0.23) and WHZ (SMD: 0.04, 95% CI: -0.13, 0.21), although showing favorable trends. MNPs were found to be associated with significant increase in diarrhea (RR: 1.04, 95% CI: 1.01, 1.06) with non-significant impacts on fever and URI.

**Conclusion:**

Our analysis of the effect of MNPs in children suggests benefit in improving anemia and hemoglobin however the lack of impact on growth and evidence of increased diarrhea requires careful consideration before recommending the intervention for implementing at scale.

## Introduction

More than 3.5 million women and children under five die each year in poor countries due to underlying undernutrition [1]. An estimated 178 million children under five are stunted and 55 million children are wasted [2]. Of these stunted children, 160 million (90%) live in just 36 countries, representing almost half of the children in those countries [2] and many of these children have concomitant micronutrient deficiencies. Deficiencies in vitamin A, iron, zinc and iodine are the most prevalent, accounting for 11% of global disease burden [3]. The World Health Organization (WHO) estimates that of the roughly two billion people suffering from micronutrient deficiencies, 85% live in resource poor settings [4]and these often occur as multiple rather than single micronutrient deficiencies [5]. The prevalence is especially high in Southeast Asia and sub-Saharan Africa.

Iron deficiency is widespread and globally about 1.62 billion people are anemic with the highest prevalence among preschool children (47%) followed by pregnant women (42%) [6]. Iodine deficiency (IDD) is a public health problem in 130 countries and affects 13% of world's population [7]. Globally about 740 million people are affected by goiter, and over two billions are considered at risk of IDD. It is estimated that one-third of the world population live in countries with a high prevalence of zinc deficiency. Clinical Vitamin A Deficiency (VAD) affects at least 2.80 million preschool children in over 60 countries, and sub clinical VAD is considered a problem for at least 251 million that includes school-age children and pregnant women [8].

Micronutrients play a critical role in cellular and humoral immune responses, cellular signaling and function, learning and cognitive functions, work capacity, reproductive health and even in the evolution of microbial virulence [9,10]. Infants, children and pregnant women have high demands for vitamins and minerals because of increased growth and metabolic requirements and yet their dietary intake often fails to meet these requirements [3,11]. In children these micronutrient deficiencies can cause anemia [12], restrict growth [13] and hamper motor and cognitive development [14] and also effect the immune function [15]. Under nutrition in children and women leaves a long term impact on population health and productivity.

Several strategies have been employed to supplement micronutrients to women and children [16-19]. These include nutrition education, dietary modification, food provision, supplementation and fortification. In the last decade point of use or home fortification of maternal and child diets has emerged to tackle the widespread micronutrient deficiencies. Multiple Micronutrient Powders (MNPs) or Sprinkles are powdered encapsulated vitamins and minerals that can be added to prepared foods with little change to the food’s taste or texture. MNPs are designed to provide the recommended daily nutrient intake of 2 or more vitamins and minerals to their target populations.

Despite the wide body of primary research on MNP interventions, there are few syntheses of the existing data. A recent Cochrane review has established that MNPs appear effective for reducing anemia and iron deficiency for children under 2 years of age [20]. We in this review have estimated the effect of these MNPs on the health of women and children. We have reviewed the available literature and evaluated the quality of included studies according to the Child Health Epidemiology Group (CHERG) adaptation of Grading of Recommendations, Assessments, Development and Education (GRADE) criteria [21].

## Methods

We systematically reviewed literature published up to November 2012 to identify studies describing the effectiveness of MNPs. Following CHERG Systematic Review Guidelines [21], we searched PubMed, Cochrane Libraries, Embase, and WHO Regional Databases to identify all published and unpublished trials. Additional studies were identified by hand searching references from included studies. Search terms included combinations of Micronutrient* OR ‘multiple micronutrient” OR “multi-vitamin” OR “multi-mineral” OR “micronutrient powder” OR MNP OR sprinkle AND Fortifi* OR “food fortifi*” OR “point of use” OR “home fortification”. No language or date restrictions were applied in the searches.

### Inclusion criteria

MNPs were identified as point-of-use powders with two or more micronutrients in their formulation. Studies were included that provided MNPs either in the home or at designated centers, using different multiple micronutrient formulations, with different dosages and duration. Studies that included supporting interventions such as nutrition education were included only if the supporting interventions were given to both the intervention and comparison groups, so that the difference between the two groups was solely of MNPs. Because of the unique nature of this intervention and a need to do a separate analysis specifically for this intervention, we excluded studies examining the impact of supplementary food provision, lipid-based supplements, micronutrient crushable tablets or foodlets, fortified milk or complementary foods and other fortified foods and beverages including fortified seasoning powders.

### Abstraction, analysis and summary measure

We abstracted data describing study identifiers and context, study design and limitations, intervention specifics and outcome effects into a standardized abstraction form for studies that met the final inclusion criteria as detailed in the CHERG Systematic Review Guidelines [21]. Outcomes of interest included hematological; anemia, hemoglobin levels, serum micronutrient levels, anthropometric; stunting, wasting, underweight, weight for age z-score (WAZ), height for age z- score (HAZ), weight for height z-score (WHZ), head circumference and morbidity; diarrhea, upper respiratory infections (URI), fever and mortality among women and children. Each study was assessed and graded according to the CHERG adaptation of the GRADE technique [21].

### Quantitative data synthesis

We conducted a meta-analysis for individual studies and pooled statistics were reported as the relative risk (RR) for categorical variables and standard mean difference (SMD) for continuous variables between the experimental and control groups with 95% confidence intervals (CI). Mantel–Haenszel pooled RR and corresponding 95% CI were reported or the DerSimonian–Laird pooled RR and corresponding 95% CI where there was an unexplained heterogeneity. All analyses were conducted using the software Review Manager 5.1. Heterogeneity was quantified by Chi^2^ and I^2^, which can be interpreted as the percentage of the total variation between studies that is attributable to heterogeneity rather than to chance, a low p-value (less than 0.1) or a large chi-squared statistic relative to its degree of freedom and I^2^ values greater than 50% were taken as substantial and high heterogeneity. In situations of high heterogeneity, causes were explored by sensitivity analysis and random effect models were used.

We summarized the evidence by outcome, including qualitative assessments of study quality and quantitative measures, according to the standard guidelines. A grade of “high”, “moderate”, “low” and “very low” was used for grading the overall evidence indicating the strength of an effect on specific health outcome according to the CHERG Rules for Evidence Review [21].

## Results

**Figure 1:**
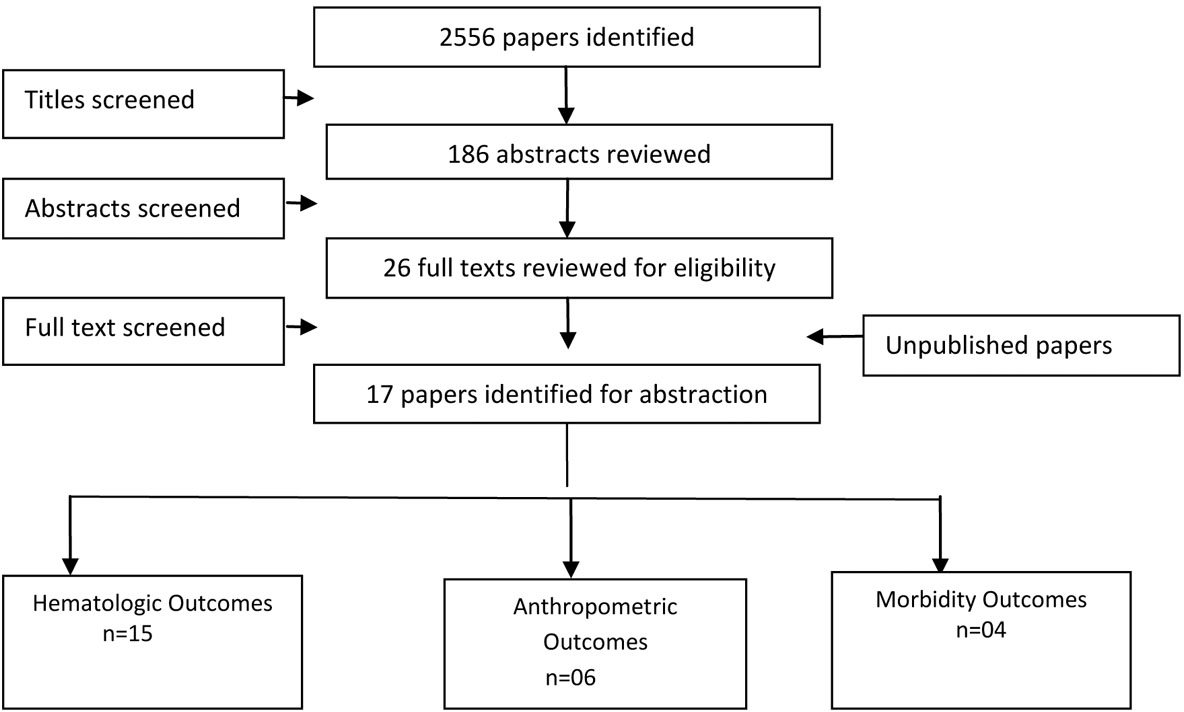
Search Flow Diagram

We identified 2556 titles from search conducted in all databases. After screening titles and abstracts, we reviewed 26 papers for the identified outcome measures of interest of which 11 papers investigated either multiple micronutrient spreads or seasonings and were excluded from this review and 17 [22-38] studies were finally selected for inclusion which evaluated the impact of MNP versus no intervention or control and reported the outcomes of interest (Figure 1). Most of the studies were done on children aged 6 months to 6 years of age, while two studies had children up to 11 years of age. All studies were conducted in developing countries. There were no studies identified which were on women and met our inclusion criteria. None of the included studies reported on the outcome of mortality. Table 1 shows the characteristics of the included studies.

**Table 1:** Characteristics of included studies

Study	Country	Target Group	MNP Composition	Duration
Adu-Afarwuah 2007	Ghana	6-12 month olds	Β-Carotene-300 µg RE, Vitamin C-50 mg, Vitamin D3-7.5 µg, Folic acid- 150 µg, Iron (Fumarate)- 12.5 mg, Zinc (Gluconate)- 5 mg	1 year
Adu-Afarwuah 2008	Ghana	6-12 month olds	Β-Carotene-300 µg RE, Vitamin C-50 mg, Vitamin D3- 7.5 µg, Folic acid- 150 µg, Iron (Fumarate)-12.5 mg, Zinc (Gluconate)- 5 mg	1 year
Agostoni 2007	Cambodia	6 month olds	Fe- 12.5mg (fumarate), Zn- 5 mg (gluconate), Vitamin C - 50mg, Vitamin A - 300mg, Vitamin D3-7.5mg, Folic acid - 150mg, Potato maltodextrins SQ to 1 g.	1 year
Kounnavong 2011	Lao People’s Democratic Republic	6-52 month olds	Vitamin A - 400μg RE, Vitamin D- 35 μg, Vitamin E - 5 mg TE, Vitamin B1, B2, B6 - each 0.5 mg, Folic acid- 150 μg, Niacin- 6 mg, Vitamin B12- 0.9 μg, Vitamin C- 30 mg, Iron- 10 mg, Zinc- 4.1 mg, Selenium- 17 μg, Copper- 0.56 mg, Iodine- 90 μg	6 months
Kumar 2007	India	7-11 year olds	Vitamin A 1500 IU/g, Vitamin B2, B6, B12 each -1 mg/g, Calcium pentothenate- 1 mg/g, Niacin -15 mg/g, Folic acid-100 mcg/g, Vitamin E -30 IU/g, Vitamin C- 30 mg/g, Iron - 10 mg/g, Lysine - 250 mg/g, Calcium - 15.63 %	1 year
Lundeen 2010	Kyrgyzistan	6-36 months old	Elemental iron (fumarate)- 12.5 mg, Vitamin A - 300 μg, Zinc (gluconate)- 5 mg, Vitamin C (ascorbic acid) - 30 mg, Folic acid- 160 μg	2 months
Macharia-Mutie 2012	Kenya	1-5 year olds	Retinyl palmitate - 100 mg RE, Cholecalciferol- 5 mg, 1-a tocopheryl acetate- 5 mg TE , Phylloquinone - 30 mg, Thiamin- 0.5 mg, Riboflavin - 0.5 mg, Pyridoxine - 0.5 mg, Folic acid - 90 mg, Niacin - 6 mg, Vitamin B-12 - 0.9 mg, Vitamin C - 60 mg, Iron (as NaFeEDTA) - 2.5 mg, Zinc - 2.5 mg, Selenium - 17 mg, Copper - 0.34 mg, Iodine- 30 mg	4 months
Menon 2007	Haiti	9-24 month olds	Iron- 12.5 mg, Zinc- 5mg, Vitamin A- 400mg, Folic acid - 160mg, Vitamin C- 30mg	2 months
Osei 2010	India	6-10 year olds	Iron (NaFeEDTA)- 10 mg, Vitamin A (retinyl acetate)- 375 mg, Zinc (zinc gluconate) -4.2 mg, Folic acid - 225 mg, Iodine (potassium iodide) - 90 mg, Vitamin C (ascorbic acid) - 26.25 mg, Thiamine (thiamine mononitrate)- 0.68 mg, Riboflavin- 0.68 mg, Niacin (nicotinamide) - 9 mg, Vitamin B-12- 1.35 mg, Vitamin B-6- 0.75 mg, Vitamin D (ergocalciferol) - 3.75 mg, Vitamin E - 5.25 mg, Copper [CuSO4.(H2O)5]- 0.45 mg	8 months
Sharieff 2006	Pakistan	6-12 month olds	Zinc gluconate- 5 mg, Ferrous fumarate - 30 mg, Vitamin C - 50 mg, Vitamin A - 300 mg, Vitamin D3 - 7.5 mg, Folic acid- 150 mg	2 months
Varma 2007	India	36-66 month olds	Ferrous fumarate- 14 mg, Vitamin A- 500 IU, Folic acid- 0.05 mg	6 months
Giovannini 2006	Cambodia	6 month olds	Fe (iron II fumarate) - 12.5 mg, Zn (gluconate) - 5 mg, Vitamin C - 50 mg, Vitamin A - 300 µg, Vitamin D3 - 7.5 µg, Folic acid 50- 150 µg, Potato maltodextrins - SQ to 1 g	1 year
Sharieff 2007	China	3-6 years	Iron (ferrous fumarate)- 30 mg, Zinc gluconate- 5mg, Vitamin C- 50 mg, Vitamin A- 300 mg, Vitamin D3 - 7.5mg, Folic acid- 150mg	3 months
Suchdev 2010	Kenya	6-35 month olds	Ferrous fumarate- 12.5mg, Vitamin A- 375 µg, Zinc- 5 mg, Folic acid- 150 µg, Vitamin C-35 mg, Vitamin D3 - 5 µg, Vitamin E- 6 mg, Niacin- 6 mg, Copper- 0.6 mg, Iodine - 50 µg, Thiamine, riboflavin and vitamin B-6 - 0.5 mg, Vitamin B-12- 0.9 mg	1 year
Jack 2012	Cambodia	6 month olds	Iron (ferrous fumarate) - 12.5 mg, Zinc gluconate - 10 mg, Vitamin A - 300μg, Iodine - 90 μg, Vitamin B1 - 0.5 mg, Vitamin B2 - 0.5 mg, Vitamin B6 - 0.5 mg, Vitamin B12 - 0.9 μg, Niacin - 6 mg, Folate, folic acid - 160 μg, Vitamin C - 30 mg, Copper - 0.3 mg, Vitamin D - 5 μg, Vitamin E - 6 IU	18 months
Bhutta (unpublished)	Pakistan	6-18 months	Ferrous fumarate- 12.5 mg, Vitamin C - 50 mg, Vitamin A (retinol acetate)- 300 μg, Vitamin D - 5 μg, Folic acid - 150 μg, Zinc gluconate- 10 mg	24 months

**Figure 2:**
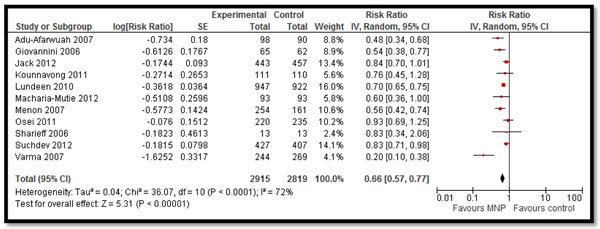
Forest Plot for the impact of MNPs on anemia in children

**Figure 3:**
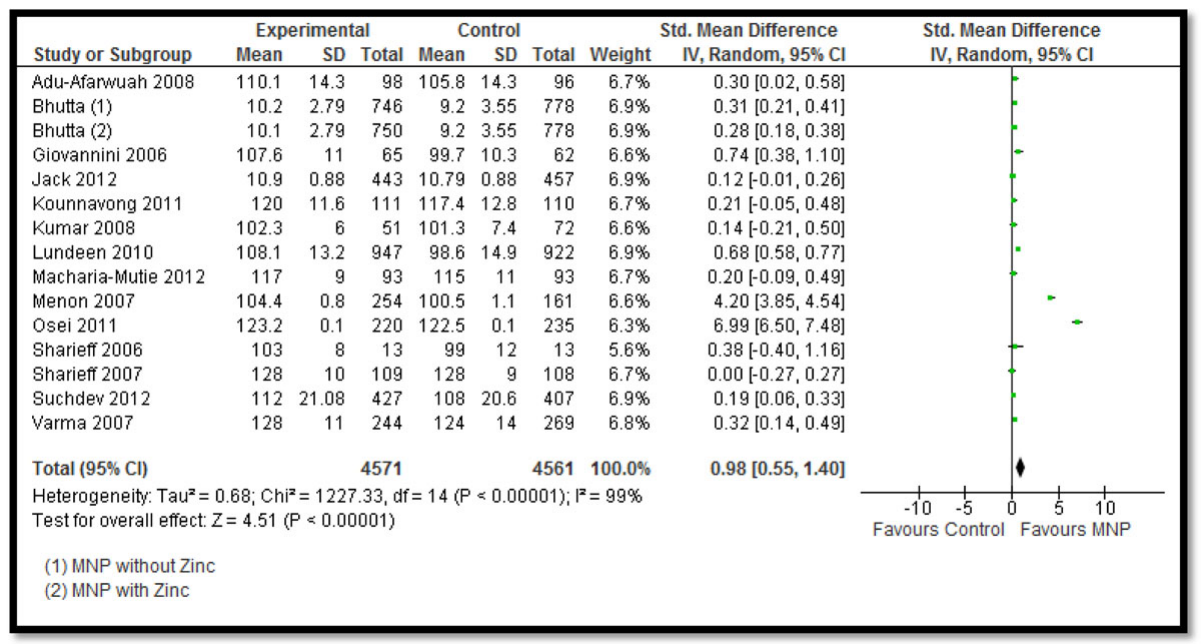
Forest Plot for the impact of MNPs on hemoglobin in children

In Table 2 and 3, we report the quality assessment of studies by outcomes. All the evidence was of moderate outcome specific quality. For the hematologic indicators (Table 2), the findings were based on 15 studies. MNPs significantly reduced the prevalence of anemia by 34% (RR: 0.66, 95% CI: 0.57-0.77) (Figure 2), iron deficiency anemia by 57% (RR: 0.43, 95% CI: 0.35-0.52) and retinol deficiency by 21% (RR: 0.79, 95% CI: 0.64, 0.98). It also significantly improved the hemoglobin levels (SMD: 0.98, 95% CI: 0.55-1.40) (Figure 3). MNPs did not show a significant improvement in serum ferritin concentration and zinc deficiency.

**Table 2:** Quality Assessment by Hematologic Outcome

	Quality Assessment					Summary of Findings		
				Directness		No of events		

No of studies	Design	Limitations	Consistency	Generalizability to population of interest	Generalizability to intervention of interest	Intervention	Control	RR / SMD (95% CI)

**Anemia: *****Moderate outcome specific quality of evidence***								

Eleven	RCT	Significant heterogeneity, random effect model used	Six of ten studies suggest benefit	All studies from the developing countries	The duration of the studies ranged from 2-12 months and the age of the children from 6 months to 10 years.	1081	1443	RR: 0.66 [0.57, 0.77]

**Iron deficiency Anemia: *****Moderate outcome specific quality of evidence***								

Seven studies (six data sets)	RCT	Significant heterogeneity, random effect model used	Four of six studies suggest benefit	All studies from the developing countries	The duration of the studies ranged from 2-12 months	404	986	RR: 0.43 [0.35, 0.52]

**Hemoglobin: *****Moderate outcome specific quality of evidence***								

Fourteen studies (Fifteen data sets)	RCT	Significant heterogeneity, random effect model used	Nine studies suggest benefit	All studies from the developing countries	Studies ranged in duration from 2-12 months.	4571	3783	SMD: 0.98 [0.55, 0.40]

**Serum Zinc: *****Moderate outcome specific quality of evidence***								

Three	RCT	Significant heterogeneity so a random effect model used	One study suggested benefit	All studies from developing countries	One study was conducted in school	761	788	SMD: -0.22 [-0.52, 0.09]

**Serum Retinol: *****Moderate outcome specific quality of evidence***								

Two	RCT	Significant heterogeneity so a random effect model used	One study suggest benefit	Both studies from India	Study duration ranged from 6-8 months.	464	504	SMD: 1.66 [-1.60, 4.92]

**Serum Ferritin: *****Moderate outcome specific quality of evidence***								

Four	RCT	Significant heterogeneity, random effect model used	Three studies suggest benefit	All studies from developing countries	Studies ranged in duration from 6 months to 12 months	850	884	SMD: 1.78 [-0.31, 3.88]

**Zinc Deficiency: *****Moderate outcome specific quality of evidence***								

Two	RCT	No significant heterogeneity, fixed effect model used	None of the study suggests benefit	All studies from the developing countries	The study duration ranged from 6-8 months.	258	272	RR: 1.02 [0.87, 1.19]

**Retinol Deficiency: *****Moderate outcome specific quality of evidence***								

Three	RCT	No significant heterogeneity, fixed effect model used	None of the study suggests benefit	All studies from the developing countries	The study duration ranged from 6-12 months.	111	145	RR: 0.79 [0.64, 0.98]

**Table 3:** Quality assessment by anthropometric and morbidity outcomes

**Stunting: *****Moderate outcome specific quality of evidence***								
Two study (three data sets)	RCT	No significant heterogeneity, fixed effect model used	None of the study suggests benefit	All studies from the developing countries		810	838	RR: 0.92 [0.81, 1.04]

**Wasting: *****Moderate outcome specific quality of evidence***								

Two study (three data sets)	RCT	No significant heterogeneity, fixed effect model used	None of the study suggests benefit	All studies from the developing countries		263	234	RR: 1.13 [0.91, 1.40]

**Underweight: *****Moderate outcome specific quality of evidence***								

Three studies (four data sets)	RCT	No significant heterogeneity, fixed effect model used	None of the studies suggest significant benefit	All the studies from developing countries	All studies included more than 3 micronutrients. The studies ranged in duration from 4-12 months.	671	679	RR: 0.96 [0.83, 1.10]

***HAZ: Moderate outcome specific quality of evidence***								

Three	RCT	No significant heterogeneity, fixed effect model used	None of the studies suggest significant benefit	All studies are from Africa	All studies included more than 3 micronutrients. The studies ranged in duration from 6-12 months.	271	253	SMD: 0.04 [-0.13, 0.22]

***WAZ: Moderate outcome specific quality of evidence***								

Three	RCT	No significant heterogeneity, fixed effect model used	None of the studies suggest significant benefit	All studies from Africa	All studies included more than 3 micronutrients. The studies ranged in duration from 6-12 months.	271	253	SMD: 0.05 [-0.12, 0.23]

**WHZ: *****Moderate outcome specific quality of evidence***								

Three	RCT	No significant heterogeneity, fixed effect model used	None of the studies suggest significant benefit	All studies from Africa	All studies included more than 3 micronutrients. The studies ranged in duration from 6-12 months.	271	253	SMD: 0.04 [-0.13, 0.21]

**Diarrhea: *****Moderate outcome specific quality of evidence***								

Four studies (five data sets)	RCT	No significant heterogeneity, fixed effect model used	Direction of evidence is consistent across studies	All the studies from developing countries	Number of micronutrients ranged from 5-15. Duration varied from 2-12 months. One of the studies targeted children in school.	1692	1679	RR: 1.04 [1.01, 1.06]

**Recurrent Diarrhea: *****Moderate outcome specific quality of evidence***								

One	RCT	Only one study		Study was conducted in Cambodia	Study was conducted over a 12 month period	1	0	RR: 2.86 [0.12, 69.00]

***URI: Moderate outcome specific quality of evidence***								

Two	RCT	No significant heterogeneity, fixed effect model used	None of the studies suggest benefit	All the studies from developing countries	Number of micronutrients ranged from 5-14. Duration varied from 8-12 months. One of the studies targeted children in school.	30	26	RR: 1.17 [0.71, 1.92]

***Fever: Moderate outcome specific quality of evidence***								

One	RCT	Only one study		Study was conducted in India		41	41	RR: 1.03 [0.70, 1.51]

For the anthropometric outcomes (Table 3), data was pooled for six studies. MNPs did not show a significant improvement in any of the anthropometric outcomes including stunting (RR: 0.92, 95% CI: 0.81, 1.04), wasting (RR: 1.13, 95% CI: 0.91, 1.40), underweight (RR:0.96, 95% CI: 0.83, 1.10), HAZ (SMD: 0.04, 95% CI: -0.13, 0.22), WAZ (SMD: 0.05, 95% CI: -0.12, 0.23) and WHZ (SMD: 0.04, 95% CI: -0.13, 0.21), although showing favorable trends, as the direction of effect was on the positive side although non-significant.

**Figure 4:**
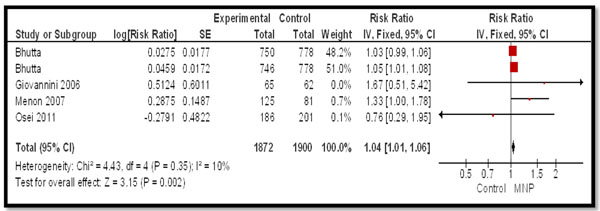
Forest Plot for the impact of MNPs on diarrhea in children

For the morbidity outcomes (Table 3), data from four studies was pooled. MNPs were associated with significant increase in the incidence of diarrhea (RR: 1.04, 95% CI: 1.01, 1.06) (Figure 4), while there was no significant rise in recurrent diarrhea (RR: 2.86, 95% CI: 0.12-69.0), fever (RR: 1.03, 95% CI: 0.70, 1.51) and URI (RR: 1.17, 95% CI: 0.71, 1.92).

### Recommendation for the LiST model

Of the outcomes assessed for the effect of MNPs in children, we applied the CHERG rules for evidence review to these outcomes. There was no data on mortality and the evidence on anthropometric outcomes is weak. With the current available evidence, we suggest that MNPs in children is associated with a 34% decrease in the incidence of anemia. The evidence of increased diarrhea suggests careful evaluation of the associated risks.

## Discussion

In this systematic review our objective was to summarize the effect of MNPs on the health outcomes of women and children. We did not find any study reporting outcomes on women and seventeen studies were included that reported on various outcomes on children. The studies contributing data in this review were conducted in developing countries hence increasing the generalizability of the studies to children in low and middle income countries with the highest undernutrition rates. Most of the studies were effectiveness trials evaluating the impact of MNPs in community settings. All of the studies were on children less than six years of age, except two studies [30,39] that included children over 6 years of age although the subgroup analysis for children under five did not show any difference in the findings. Clinical heterogeneity was observed due to variations in type of intervention (number of micronutrients used ranged from 3 to 15), duration of the intervention (2-24 months), target population and different time intervals for follow-up. All the MNPs used contained iron in their composition.

The intervention was mostly reported to be acceptable by the mothers and children and there was no major loss to follow-up reported due to the intervention in any of the included studies. There have been no adverse events identified by any study except one [38] that reported increased diarrhea in the intervention group compared to control.

This review shows that MNPs raise serum hemoglobin levels and reduce anemia significantly, but the evidence on growth is weak, as relatively few studies have evaluated this outcome. Improved hemoglobin and anemia status could be attributable to the iron component in all the MNPs used. Some studies have reported benefits on other developmental outcomes like walking by 12 months but not on growth [22]. This could be due to relatively shorter duration of the intervention to show actual long term impacts. These findings also suggest that multiple micronutrient interventions alone might not improve growth outcomes. To ensure long term impacts and sustainability, health education that aims to modify food habits would be necessary to improve child growth rates. Also, if the intervention initiation coincides with the child’s diet transition from breast feeding to complementary feeding, the results may show improved growth.

The finding of significantly increased diarrhea is potentially alarming. It is mainly based on the significant increase in diarrhea observed in one large trial [38]. The association between increased diarrhea with iron supplementation is well recognized in the literature and is also reported in a review on iron supplementation by Gera [40]. However, our finding of excess morbidity and negligible growth benefit cannot be ignored in settings where large scale use of MNPs is being considered. The increased diarrhea burden could be one of the potential explanations for reduced growth benefits of MNPs.

The evidence is weak for any effect of MNPs on growth, as there were very few studies pooled for each outcome. More research is needed and studies need to report the outcomes of stunting, wasting, morbidity and mortality consistently to strengthen the evidence and evaluate its actual impact on growth and morbidity. A major research gap identified was that there were no studies evaluating the impact on women as all the studies targeted children only.

## Conclusion

Our analysis of the effect of MNPs in children suggests benefit in improving anemia and hemoglobin however there is lack of impact on growth. Evidence of increased diarrhea requires careful consideration before recommending the intervention for implementation at scale.

## Competing interests

We do not have any financial or non-financial competing interests for this review.

## Authors' contributions

Dr. ZAB was responsible for designing the review and coordinating the review. RAS, CM and JKD were responsible for: data collection, screening the search results, screening retrieved papers against inclusion criteria, appraising quality of papers, abstracting data from papers, entering data into RevMan, analysis and interpretation of data and writing the review. ZAB and RAS critically reviewed and modified the manuscript.

## Declarations

The publication costs for this supplement were funded by a grant from the Bill & Melinda Gates Foundation to the US Fund for UNICEF (grant 43386 to "Promote evidence-based decision making in designing maternal, neonatal, and child health interventions in low- and middle-income countries”). The Supplement Editor is the principal investigator and lead in the development of the Lives Saved Tool (LiST), supported by grant 43386. He declares that he has no competing interests.

This article has been published as part of *BMC Public Health* Volume 13 Supplement 3, 2013: The Lives Saved Tool in 2013: new capabilities and applications. The full contents of the supplement are available online at http://www.biomedcentral.com/bmcpublichealth/supplements/13/S3.

## References

[B1] WHO Severe malnutrition: report of a consultation to review current literature 2000 Geneva: World Health Organization

[B2] Bhutta ZA Micronutrient needs of malnourished children Curr Opin Clin Nutr Metab Care 2008 11 3 309 314 10.1097/MCO.0b013e3282fbf5a0 18403929

[B3] Black RE Allen LH Bhutta ZA Caulfield LE De Onis M Ezzati M Mathers C Rivera J Maternal and child undernutrition: global and regional exposures and health consequences Lancet 2008 371 9608 243 260 10.1016/S0140-6736(07)61690-0 18207566

[B4] WHO World health report 2000 Geneva: World Health Organization

[B5] Abu-Saad K Fraser D Maternal nutrition and birth outcomes Epidemiologic Reviews 2010 32 1 5 25 10.1093/epirev/mxq001 20237078

[B6] Benoist B McLean E Egll I Cogswell M Worldwide prevalence of anaemia 1993-2005: WHO global database on anaemia Worldwide prevalence of anaemia 1993-2005: WHO global database on anaemia 2008

[B7] Vir SC Current status of iodine deficiency disorders (IDD) and strategy for its control in India Indian journal of pediatrics 2002 69 7 589 596 10.1007/BF02722687 12173699

[B8] Stephenson LS Latham MC Ottesen EA Global malnutrition Parasitology 2000 121 Suppl S5 S22 11386691 10.1017/s0031182000006478

[B9] Guerrant RL Lima AAM Davidson F Micronutrients and infection: interactions and implications with enteric and other infections and future priorities Journal of Infectious Diseases 2000 182 Supplement 1 S134 S138 10944495 10.1086/315924

[B10] Kapil U Bhavna A Adverse effects of poor micronutrient status during childhood and adolescence Nutr Rev 2002 60 5 Pt 2 S84 S90 12035866 10.1301/00296640260130803

[B11] Black RE Micronutrients in pregnancy British Journal of Nutrition 2001 85 Suppl 2 S193 197 11509110 10.1079/bjn2000314

[B12] Le HT Brouwer ID Verhoef H Nguyen KC Kok FJ Anemia and intestinal parasite infection in school children in rural Vietnam Asia Pacific Journal of Clinical Nutrition 2007 16 4 716 723 18042534

[B13] Lawless JW Latham MC Stephenson LS Kinoti SN Pertet AM Iron supplementation improves appetite and growth in anemic Kenyan primary school children The Journal of nutrition 1994 124 5 645 8169656 10.1093/jn/124.5.645

[B14] Black RE Zinc deficiency, infectious disease and mortality in the developing world Journal of Nutrition 2003 133 5 Suppl 1 1485S 1489S 12730449 10.1093/jn/133.5.1485S

[B15] Thurnham DI Micronutrients and immune function: some recent developments Journal of clinical pathology 1997 50 11 887 891 10.1136/jcp.50.11.887 9462235 PMC500310

[B16] Best C Neufingerl N Del Rosso JM Transler C van den Briel T Osendarp S Can multi micronutrient food fortification improve the micronutrient status, growth, health, and cognition of schoolchildren? A systematic review Nutrition Reviews 2011 69 4 186 204 10.1111/j.1753-4887.2011.00378.x 21457264

[B17] Bhutta ZA Ahmed T Black RE Cousens S Dewey K Giugliani E Haider BA Kirkwood B Morris SS Sachdev HPS What works? Interventions for maternal and child undernutrition and survival Lancet 2008 371 9610 417 440 10.1016/S0140-6736(07)61693-6 18206226

[B18] Dewey KG Yang Z Boy E Systematic review and meta analysis of home fortification of complementary foods Maternal Child Nutrition 2009 5 4 283 321 10.1111/j.1740-8709.2009.00190.x

[B19] Serdula M Maximizing the impact of flour fortification to improve vitamin and mineral nutrition in populations Food and nutrition bulletin 2010 31 1 Suppl S86 93 20629355 10.1177/15648265100311s108

[B20] De-Regil LM Suchdev PS Vist GE Walleser S Peña-Rosas JP Home fortification of foods with multiple micronutrient powders for health and nutrition in children under two years of age Cochrane Database Syst Rev 2011 9 CD008959 10.1002/14651858.CD008959.pub2 21901727

[B21] Walker N Fischer-Walker C Bryce J Bahl R Cousens S Standards for CHERG reviews of intervention effects on child survival International journal of epidemiology 2010 39 suppl 1 i21 i31 20348122 10.1093/ije/dyq036 PMC2845875

[B22] Adu-Afarwuah S Lartey A Brown KH Zlotkin S Briend A Dewey KG Randomized comparison of 3 types of micronutrient supplements for home fortification of complementary foods in Ghana: effects on growth and motor development American Journal of Clinical Nutrition 2007 86 2 412 420 17684213 10.1093/ajcn/86.2.412

[B23] Adu-Afarwuah S Lartey A Brown KH Zlotkin S Briend A Dewey KG Home fortification of complementary foods with micronutrient supplements is well accepted and has positive effects on infant iron status in Ghana American Journal of Clinical Nutrition 2008 87 4 929 938 18400716 10.1093/ajcn/87.4.929

[B24] Agostoni C Riva E Giovannini M Functional ingredients in the complementary feeding period and long-term effects Nestle Nutrition Workshop Series Paediatric Programme 2007 60 123 135 discussion 135-128 17664901 10.1159/000106365

[B25] Kounnavong S Sunahara T Mascie-Taylor CG Hashizume M Okumura J Moji K Boupha B Yamamoto T Effect of daily versus weekly home fortification with multiple micronutrient powder on haemoglobin concentration of young children in a rural area, Lao People's Democratic Republic: a randomised trial Nutrition Journal 2011 10 129 10.1186/1475-2891-10-129 22111770 PMC3266642

[B26] Kumar MV Rajagopalan S Multiple micronutrient fortification of salt and its effect on cognition in Chennai school children Asia Pacific Journal of Clinical Nutrition 2007 16 3 505 511 17704033

[B27] Lundeen E Schueth T Toktobaev N Zlotkin S Hyder SM Houser R Daily use of Sprinkles micronutrient powder for 2 months reduces anemia among children 6 to 36 months of age in the Kyrgyz Republic: a cluster-randomized trial Food & Nutrition Bulletin 2010 31 3 446 460 20973465 10.1177/156482651003100307

[B28] Macharia-Mutie CW Moretti D Van den Briel N Omusundi AM Mwangi AM Kok FJ Zimmermann MB Brouwer ID Maize porridge enriched with a micronutrient powder containing low-dose iron as NaFeEDTA but not amaranth grain flour reduces anemia and iron deficiency in Kenyan preschool children Journal of Nutrition 2012 142 9 1756 1763 10.3945/jn.112.157578 22810982

[B29] Menon P Ruel MT Loechl CU Arimond M Habicht JP Pelto G Michaud L Micronutrient Sprinkles reduce anemia among 9- to 24-mo-old children when delivered through an integrated health and nutrition program in rural Haiti Journal of Nutrition 2007 137 4 1023 1030 17374671 10.1093/jn/137.4.1023

[B30] Osei AK Rosenberg IH Houser RF Bulusu S Mathews M Hamer DH Community-level micronutrient fortification of school lunch meals improved vitamin A, folate, and iron status of schoolchildren in Himalayan villages of India Journal of Nutrition 2010 140 6 1146 1154 10.3945/jn.109.114751 20410083

[B31] Sharieff W Yin SA Wu M Yang Q Schauer C Tomlinson G Zlotkin S Short-term daily or weekly administration of micronutrient Sprinkles has high compliance and does not cause iron overload in Chinese schoolchildren: a cluster-randomised trial Public Health Nutrition 2006 9 3 336 344 10.1079/PHN2006841 16684385

[B32] Varma JL Das S Sankar R Mannar MG Levinson FJ Hamer DH Community-level micronutrient fortification of a food supplement in India: a controlled trial in preschool children aged 36-66 mo American Journal of Clinical Nutrition 2007 85 4 1127 1133 17413115 10.1093/ajcn/85.4.1127

[B33] Giovannini M Sala D Usuelli M Livio L Francescato G Braga M Radaelli G Riva E Double-blind, placebo-controlled trial comparing effects of supplementation with two different combinations of micronutrients delivered as sprinkles on growth, anemia, and iron deficiency in Cambodian infants Journal of pediatric gastroenterology and nutrition 2006 42 3 306 312 10.1097/01.mpg.0000189363.07040.4b 16540800

[B34] Sharieff W Yin S Wu M Yang Q Schauer C Tomlinson G Zlotkin S Short-term daily or weekly administration of micronutrient Sprinkles has high compliance and does not cause iron overload in Chinese schoolchildren: a cluster-randomised trial Public Health Nutr 2006 9 3 336 344 10.1079/PHN2006841 16684385

[B35] Suchdev PS Ruth L Obure A Were V Ochieng C Ogange L Owuor M Ngure F Quick R Juliao P Monitoring the marketing, distribution, and use of Sprinkles micronutrient powders in rural western Kenya Food & Nutrition Bulletin 2010 31 Supplement 2 168 178 20715601 10.1177/15648265100312S209

[B36] Suchdev PS Ruth LJ Woodruff BA Mbakaya C Mandava U Flores-Ayala R Jefferds MED Quick R Selling Sprinkles micronutrient powder reduces anemia, iron deficiency, and vitamin A deficiency in young children in Western Kenya: a cluster-randomized controlled trial The American journal of clinical nutrition 2012 95 5 1223 1230 10.3945/ajcn.111.030072 22492366 PMC4697950

[B37] Jack SJ Ou K Chea M Effect of micronutrient Sprinkles on reducing anemia: a cluster-randomized effectiveness trial Arch Pediatr Adolesc Med 2012 166 9 842 850 10.1001/archpediatrics.2012.1003 22801933

[B38] Soofi S Cousens S Iqbal SP Akhund T Ahmed I Zaidi AKM Bhutta ZA Effect of provision of daily zinc and iron with several micronutrients on growth and morbidity among young children in Pakistan: a cluster-randomised trial Lancet 2013 382 9886 29 40 10.1016/S0140-6736(13)60437-7 23602230

[B39] Vinod Kumar M Rajagopalan S Trial using multiple micronutrient food supplement and its effect on cognition Indian Journal of Pediatrics 2008 75 7 671 678 10.1007/s12098-008-0127-1 18716734

[B40] Gera T Sachdev HPS Effect of iron supplementation on incidence of infectious illness in children: systematic review BMJ 2002 325 7373 1142 10.1136/bmj.325.7373.1142 12433763 PMC133452

